# Diverse hosts, diverse immune systems: Evolutionary variation in bat immunology

**DOI:** 10.1111/nyas.15395

**Published:** 2025-07-03

**Authors:** Daniel J. Becker, Amanda Vicente‐Santos, Ashley B. Reers, B. R. Ansil, Mika O'Shea, Caroline A. Cummings, Alicia J. Roistacher, Rita M. Quintela‐Tizon, Manuela M. T. Pereira, Juniper Rosen, Arinjay Banerjee, Hannah K. Frank

**Affiliations:** ^1^ School of Biological Sciences University of Oklahoma Norman Oklahoma USA; ^2^ Department of Ecology and Evolutionary Biology Tulane University New Orleans Louisiana USA; ^3^ Vaccine and Infectious Disease Organization Saskatoon Saskatchewan Canada; ^4^ Department of Veterinary Microbiology University of Saskatchewan Saskatoon Saskatchewan Canada; ^5^ Department of Biology University of Waterloo Toronto Ontario Canada; ^6^ Department of Laboratory Medicine and Pathobiology University of Toronto Toronto Ontario Canada; ^7^ Department of Biochemistry and Molecular Biology University of British Columbia Vancouver British Columbia Canada

**Keywords:** Chiroptera, ecoimmunology, phylogenetic comparative methods, phylogeography, SARS‐CoV‐2, white‐nose syndrome, zoonotic spillover

## Abstract

The ability of multiple bat species to host zoonotic pathogens without often showing disease has fostered a growing interest in bat immunology to discover the ways immune systems may differ between bats and other vertebrates. However, interspecific variation in immunological diversity among bats has only begun to be recognized. The order Chiroptera accounts for over 20% of all mammalian species and shows extreme diversity in a suite of correlated ecological traits, such that bats should not be expected to be immunologically homogenous. We review the ecological and evolutionary diversity of chiropteran hosts and highlight case studies emphasizing the range of immune strategies thus far observed across bat species, including responses to SARS‐CoV‐2. Next, we synthesize and propose hypotheses to explain this immunological diversity, focusing on pathogen exposure, biogeography, host energetics, and environmental stability. We then analyze immunology‐related citations across bat species to motivate discussions of key research priorities. Broad sampling is needed to remedy current biases, as only a fraction of bat species has been immunologically studied. Such work should integrate methodological advancements, in vitro and in vivo studies, and phylogenetic comparative methods to robustly test evolutionary hypotheses and understand the drivers and consequences of immunological diversity among bats.

## Graphical abstract

Bats are recognized to have distinct immune systems from other vertebrates that may allow them to host virulent pathogens without showing disease. However, these flying mammals are also incredibly diverse, such that bats should not be expected to be immunologically homogenous. We highlight the diversity of immune strategies among bat species observed to date, propose hypotheses to explain this heterogeneity, and outline critical future research priorities.

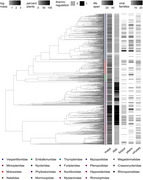

## INTRODUCTION

Over the past decades, bats have been linked to numerous spillovers of zoonotic pathogens, including viruses such as Hendra and Nipah virus, SARS‐like coronaviruses (CoVs), Marburg virus (MARV), and MERS‐like CoVs; bacteria such as *Candidatus* Bartonella mayotimonensis, *Candidatus* Bartonella rousetti, and *Candidatus* Mycoplasma haematohominis; and protozoa such as *Trypanosoma cruzi*.^1^, ^2^, ^3^, ^4^, ^5^, ^6^, ^7^, ^8^, ^9^ These spillovers, alongside observations that bats often host such pathogens without overt signs of disease, have generated substantial interest in bat immunology and understanding mechanisms of host resistance and tolerance.^10^, ^11^, ^12^, ^13^, ^14^, ^15^, ^16^ Bats are also exceptional among mammals in other ways; they are the only mammals with powered flight, are potentially resistant to cancer, and many have long lifespans for their body size.^17^, ^18^, ^19^, ^20^, ^21^ The association of many bat species with multiple pathogens and their unique adaptations have led to hypotheses about how bats, as an order, may differ in their immune system from other mammals. The *flight as fever* hypothesis posits that the elevated body temperatures bats reach during powered flight could dampen viral replication or select for viruses able to withstand the febrile responses of other mammals.^22^ However, this hypothesis has received little support,^23^, ^24^ with growing evidence suggesting that flight has likely shaped bat immunity in other ways.^25^, ^26^ For example, metabolic demands of flight generate high oxidative stress,^27^ such that bats have evolved mechanisms to withstand subsequent DNA damage while avoiding pathology by downregulating inflammatory pathways.^28^, ^29^ These adaptations have been proposed to explain why bats often tolerate intracellular infections while also being susceptible to certain extracellular infections (e.g., *Pseudogymnoascus destructans*, the fungus that causes white‐nose syndrome [WNS], which has decimated populations of multiple hibernating North American bat species).^14^, ^23^

Support for hypotheses about distinct immune adaptations of bats largely stems from a small but growing number of model systems in bat immunology.^30^, ^31^, ^32^, ^33^ However, while multiple immune adaptations are certainly present across bat species, immunological diversity within the order Chiroptera is also becoming increasingly acknowledged and characterized.^19^, ^29^, ^34^, ^35^, ^36^ In this review, we highlight the diversity of immune systems across this hyperdiverse clade of mammals, emphasizing that bats—as an order—are far from immunologically homogenous. We also synthesize proposed evolutionary hypotheses underlying this diversity and suggest future directions to test such hypotheses. We do not exhaustively summarize the state of research on bat immunology or the immune characteristics that make bats distinct from other mammals given previous reviews on these topics.^37^, ^38^, ^39^ Our objectives are for this review to serve as an entry point for immunologists to consider variation within this group of flying mammals as well as a resource for both field and comparative biologists to test central evolutionary hypotheses.

## ECOLOGICAL AND EVOLUTIONARY DIVERSITY AMONG BATS

Bats are the second largest mammalian order (after rodents), accounting for over 20% of all mammalian species. The order Chiroptera originated during the Cretaceous–Tertiary boundary, approximately 65 million years ago (mya), followed by a divergence into two monophyletic suborders: Yinpterochiroptera and Yangochiroptera.^40^, ^41^ This divergence was followed by a rapid radiation event during the early Eocene (56–47 mya), coinciding with global temperature rise and concurrent expansion of plant and insect diversity.^42^, ^43^, ^44^ Multiple, subsequent radiations, such as those of the Phyllostomidae in the Western Hemisphere (30 mya) and the Pteropodidae in the Eastern Hemisphere (25 mya), were further driven by factors including niche partitioning, novel innovations (e.g., phytophagy), and geographic isolation.^45^, ^46^ These evolutionary processes generated the remarkable diversity of bats, resulting in 1487 extant species across 21 families.^47^ Underexplored tropical regions and unclear taxonomic boundaries (e.g., cryptic species) are expected to only further increase bat global diversity.^48^, ^49^ Bats inhabit a wide variety of terrestrial habitats on every continent except for Antarctica, with some species occupying up to seven or eight distinct habitat types (e.g., *Rousettus aegyptiacus* and *Taphozous nudiventris*, respectively), as defined by the International Union for the Conservation of Nature (IUCN).^50^

**FIGURE 1:**
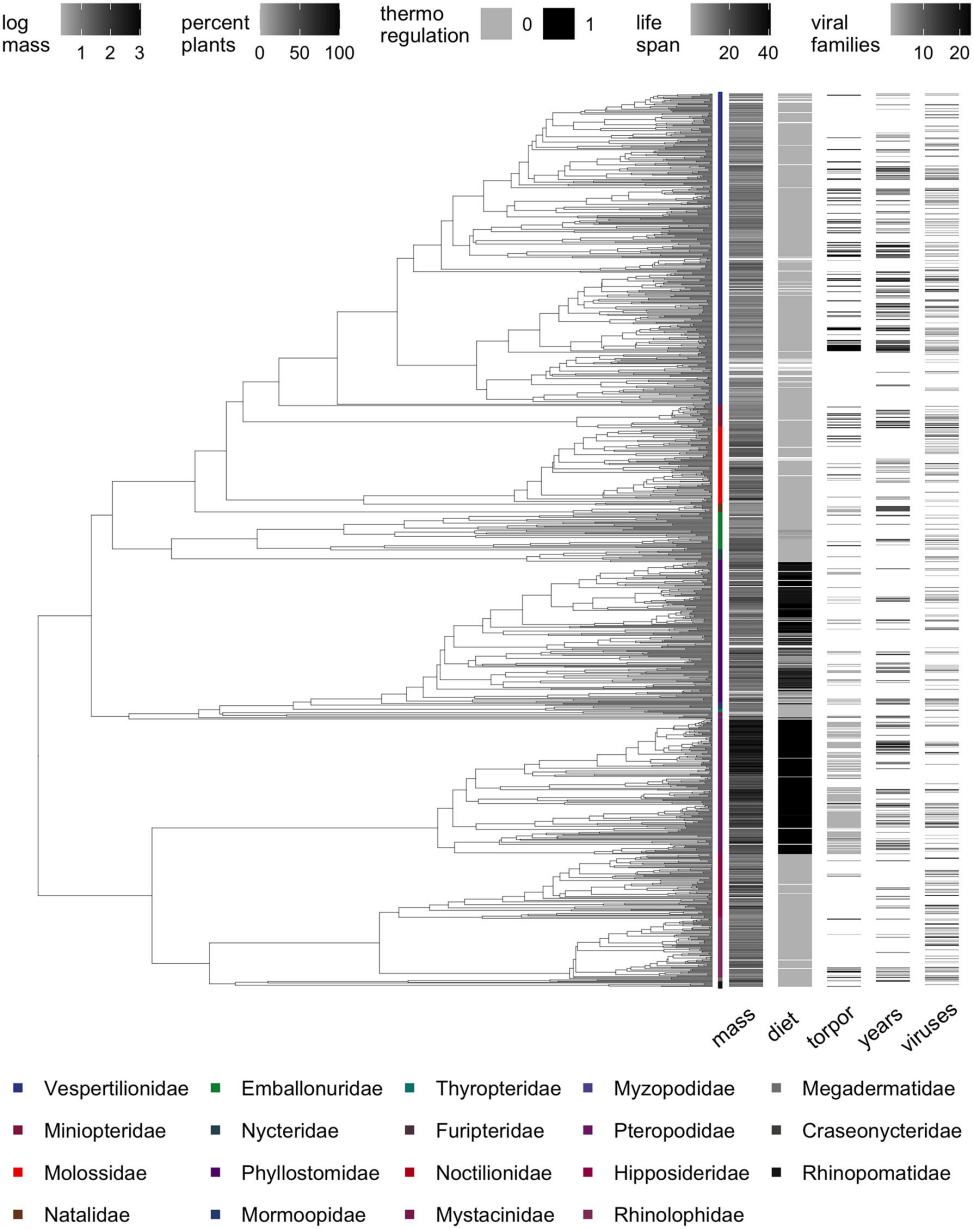
Representative axes of ecological and epidemiological variation among bat species using the most recent mammal phylogeny (1287 bat species, colored by family).^231^ Body mass, phytophagy, thermoregulation (i.e., torpor or hibernation; 1), and maximum lifespan were obtained from the COalesced Mammal dataBase of INtrinsic and Extrinsic traits (COMBINE) database of mammalian traits.^50^ Viral family richness data were obtained from the Global Virome in One Network (VIRION) database,^75^ simplified to only those records detected through sequencing or isolation, resolved by NCBI, and aligned to the tree taxonomy. Missing data are shown in white.

Bats accordingly exhibit a remarkable array of morphological (e.g., body mass), ecological (e.g., diet), and physiological adaptations (e.g., echolocation) that evolved to suit their ecological niches and life history strategies (Figure 1).^51^ For example, body mass varies over three orders of magnitude across bats, ranging from just a few grams in small insectivores (e.g., *Craseonycteris thonglongyai*, which weighs approximately 2 grams) to over a kilogram in larger frugivores (e.g., *Acerodon jubatus*).^50^ Frugivorous bats are generally larger with broader wingspans, while insectivorous bats tend to be smaller with shorter wingspans to improve agility.^52^, ^53^ The specialized facial morphologies of bats also evolved as adaptations to their diverse dietary habits, including nectarivory (e.g., *Leptonycteris yerbabuenae*), frugivory (e.g., *Pteropus medius*), insectivory (e.g., *Myotis myotis*), carnivory (e.g., *Macroderma gigas*), piscivory (e.g., *Noctilio leporinus*), and hematophagy (e.g., *Desmodus rotundus*).^54^, ^55^

Morphology and foraging ecology are only two of the multiple axes of variation among the Chiroptera. Physiological adaptations such as metabolic rates, thermoregulation mechanisms, and sensory abilities vary widely across species, allowing bats to inhabit diverse habitats (Figure 1).^50^, ^56^ For example, some bat species adjust their metabolic rate (i.e., torpor) to allow matching their activity level to environmental conditions.^57^ Hibernation, a more extreme drop in metabolic rate, is used mostly by Neartic and Paleartic bats to avoid harsh winter temperatures,^58^ but this adaptation also occurs in tropical species and has evolved multiple times in bats.^59^, ^60^ Other bat species instead undertake long‐distance latitudinal (e.g., *Tadarida brasiliensis*) or altitudinal (e.g., *Miniopterus natalensis*) migrations to escape extreme temperatures.^61^, ^62^, ^63^ This metabolic flexibility is also one of the evolutionary drivers for the exceptional longevity seen in bats as compared to other small mammals.^17^ Although bats overall have a slow life‐history strategy, species do vary along the fast–slow continuum (Figure 1).^50^, ^64^ For example, *Myotis brandtii* can live for up to 41 years,^65^ in contrast to the average bat lifespan of 15 years.^50^ Similarly, while most bat species have one breeding cycle per year with a single pup,^50^, ^66^ some species are polyestrous (e.g., *Tadarida fulminans*, multiple phyllostomids^67^, ^68^) and/or polytocous (e.g., seen mostly in the Vespertilionidae but also in other families such as the Pteropodidae^69^, ^70^).

While the order Chiroptera has been characterized as having high pathogen richness,^71^ likely due to the speciose nature of this clade,^72^ bats also vary in their pathogen associations, with most data on viruses and bacteria.^73^, ^74^, ^75^ For the former, over one‐quarter of bat species host at least one virus, with infected species hosting an average of four and up to 23 viral families (Figure 1).^75^ The propensity for some bats to host typically virulent viruses has imposed extreme selection on bat genomes for mechanisms of viral resistance (e.g., selection of antiviral effector genes and complement genes) and tolerance (e.g., regulation of inflammatory response).^15^, ^76^ However, distinct coevolutionary histories between bats and their viruses,^77^, ^78^ coupled with substantial variation in observed viral diversity among species,^75^ have likely also shaped distinct defense strategies and corresponding immune phenotypes across the chiropteran phylogeny.

## BATS ARE NOT A MONOLITH: INTERSPECIFIC VARIATION IN BAT IMMUNITY

**FIGURE 2:**
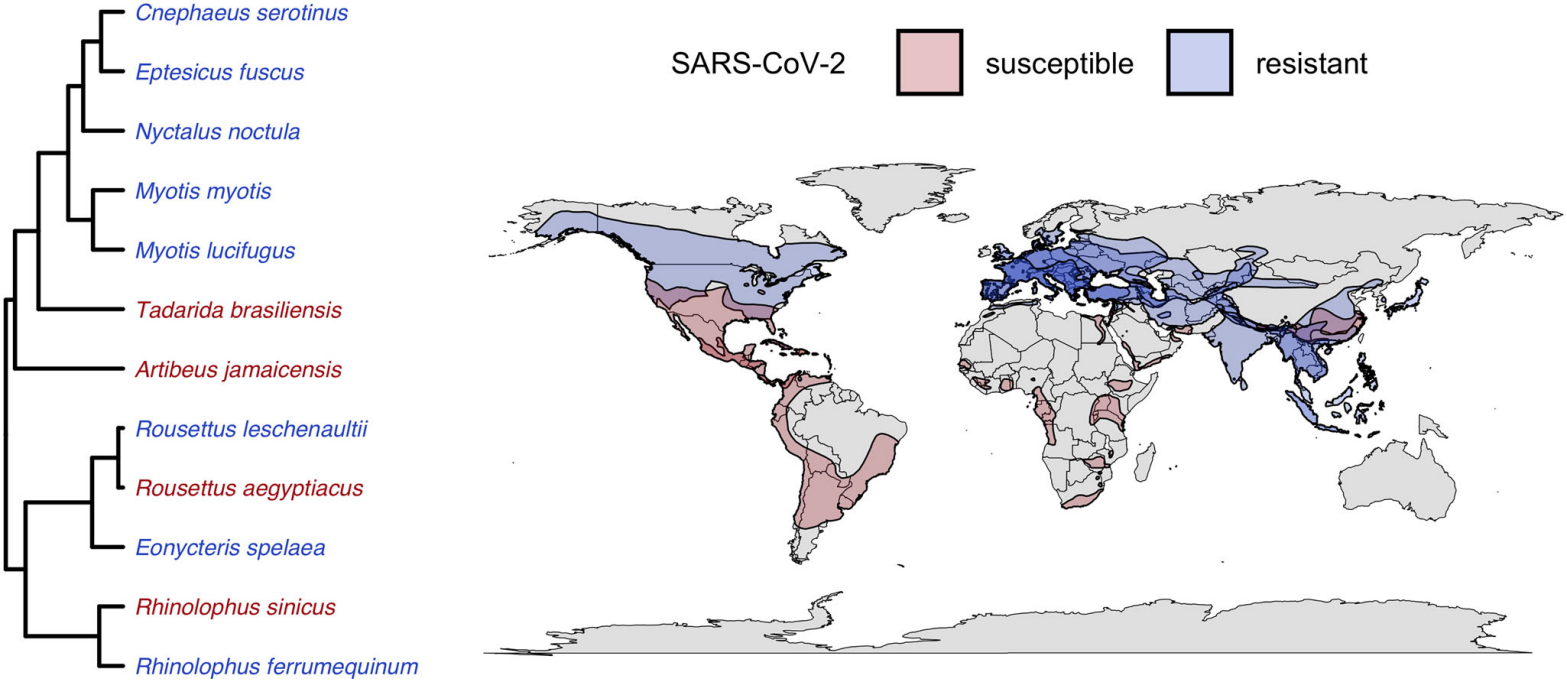
Phylogeography of bat species shown to be susceptible or resistant to SARS‐CoV‐2 infection through in vivo or in vitro challenge using the most recent mammal phylogeny,^231^ experimental data,^79^, ^80^, ^81^, ^82^, ^83^, ^84^, ^85^, ^87^,^87^, ^93^ and species distributions from the IUCN. Subgenera of the genus *Eptesicus* were recently elevated to full genus rank, such that species in the Eastern Hemisphere have been reclassified into the genus *Cnephaeus*.^279^ We note that SARS‐CoV‐2 isolates used in these experimental studies were derived from humans and thus do not represent interactions of bats or their cells with bona fide bat‐derived SARS‐CoV‐2–like viruses, such as BANAL‐236.^280^

Given the substantial diversity in morphological, ecological, and physiological traits of bats; their long coevolutionary relationships with pathogens; and variance in pathogen richness, bat immune systems are expected to be equally heterogeneous. Recent in vivo and in vitro studies have begun to reveal an array of species‐specific immune responses, shedding light on the distinct immune strategies that bat species use against their viral pathogens. As one key example, in the case of SARS‐CoV‐2 in vivo infections, both *Eptesicus fuscus* and *Myotis lucifugus* were resistant, while *Tadarida brasiliensis* was susceptible but likely not competent for onward transmission.^79^, ^80^, ^81^, ^82^ Similarly, *Rousettus aegyptiacus* challenged with SARS‐CoV‐2 were susceptible but had transient infections, with limited bat–bat transmission.^83^, ^84^ Other in vitro studies have shown that *Myotis myotis*, *Eptesicus serotinus*, *Tadarida brasiliensis*, and *Nyctalus noctula* wing cells were not permissive to SARS‐CoV‐2 due to low expression of the angiotensin‐converting enzyme 2 (ACE2) receptor or to poor interactions between ACE2 and the viral S protein.^85^ ACE2 receptor sequences and the selection acting on them also vary between bat species, further shaping differences in SARS‐CoV‐2 susceptibility.^86^ Additionally, intestinal organoids of *Rhinolophus sinicus* were susceptible to SARS‐CoV‐2 and sustained viral replication^87^, while fibroblasts of *Rhinolophus ferrumequinum* were resistant to infection.^88^ Intestinal organoids of *Rousettus leschenaultii* and airway epithelial cells of *Eonycteris spelaea* were also resistant to infection,^89^ ^,^ ^90^ while both intestinal organoids and in vivo challenge of *Artibeus jamaicensis* show this species is susceptible but does not support SARS‐CoV‐2 replication.^91^, ^92^ With the caveat that these cell lines only represent select tissue types, and additional cell lines from other organs could yield different results with SARS‐CoV‐2 challenge, these in vivo and in vitro case studies highlight substantial species‐level heterogeneity in bat susceptibility and suitability for SARS‐CoV‐2 infection, even in species in the same genus (Figure 2). Importantly, the bat species involved in these diverse challenges originate from both hemispheres and include susceptible and resistant species in multiple families. This suggests differences in susceptibility are unlikely to stem only from coevolutionary history as the current repertoire of sarbecoviruses and their known bat hosts are restricted to the Eastern Hemisphere, largely in the Paleartic and Indomalayan regions.^93^

Interspecific differences in infection response have been observed for other viruses. *Eidolon helvum* cells were refractory to Ebola virus (EBOV) entry due to a single mutation in the filovirus receptor, Niemann‐Pick C1; species without this mutation are likely susceptible to filovirus entry.^94^ Further, *Rousettus aegyptiacus* were susceptible to MARV but resistant to EBOV, highlighting that even closely related viruses (both within the *Filoviridae*) can have different outcomes in the same species.^95^ In the case of rabies virus (RABV), outcomes can vary both across and within species, highlighting the complex nature of the relationships between bat immunity and infection.^96^, ^97^, ^98^ Work on RABV has shown especially interesting differences in adaptive immunity. Following RABV infection, some *Eptesicus fuscus* failed to seroconvert and succumbed to infection.^99^ In contrast, some *Desmodus rotundus* vaccinated against and challenged with RABV survived despite not producing detectable antibody titers.^100^

Given the logistical challenges of in vivo or in vitro experiments using pathogens, the use of pathogen‐associated molecular patterns (PAMPs) that instead stimulate a more general acute phase response without true infection has suggested additional interspecific differences in bat immune systems. For example, in response to a lipopolysaccharide (LPS) challenge to mimic a bacterial infection, *Molossus molossus* had no detectable inflammation, while *Desmodus rotundus* experienced pronounced leukocytosis and behavioral changes.^101^, ^102^ In contrast, *Carollia perspicillata* challenged with LPS also displayed no fever or leukocytosis but did show decreased food intake and lost body mass.^103^ Importantly, these studies used similar doses of LPS,^101^, ^102^, ^103^ facilitating interspecies comparisons—although methodological differences can often vary substantially across studies using PAMPs.^104^ *Desmodus* and *Carollia* are both in the family Phyllostomidae, while *Molossus* is in the Molossidae, suggesting evolutionary and intrafamily effects that could stem from species differences in ecology or life history. Similarly, while an in vitro challenge with polyinosinic:polycytidylic acid (polyI:C) to mimic an RNA virus infection upregulates similar genes related to cytokine and inflammatory responses across phylogeographically diverse bats (i.e., *Rousettus aegyptiacus*, *Pipistrellus kuhlii*, *Eptesicus fuscus*, *Cnephaeus* *nilsonii*), species‐specific differences were also observed (e.g., between *Rousettus aegyptiacus* and *Pipistrellus kuhlii*).^105^, ^106^ Such challenges have also revealed intrafamily differences in the bat antiviral response. For example, constitutive expression of interferon alpha (IFN‐α) has been observed in *Pteropus alecto* tissues but not in *Rousettus leschenaultii* kidney cells, despite both species belonging to the family Pteropodidae; stimulation with polyI:C increased IFN‐α expression in the latter but not the former species.^31^, ^107^

Beyond viral and bacterial infections, bats also show varied susceptibility to fungal pathogens, notably *Pseudogymnoascus destructans*. The highly susceptible Nearctic *Myotis lucifugus* mounts a substantial transcriptomic response to infection, upregulating leukocyte activation and inflammatory pathways, whereas the tolerant Palearctic *Myotis myotis* has a nearly undetectable transcriptional response.^108^ The less‐susceptible Nearctic *Eptesicus fuscus* exhibits a similar gene expression profile to *Myotis lucifugus* but instead mounts a localized, nonsystemic response. Across these three host–pathogen contexts, the fungal transcriptome is notably consistent, highlighting bat species‐level differences that drive WNS outcomes.^109^

A larger body of work on immune profiles of wild bats at baseline has also revealed immunological differences among species, although such patterns are more difficult to interpret given the unknowns about pathogen exposure history.^110^ For example, white blood cell counts varied substantially across a Neotropical bat community in Costa Rica, with larger bat species and carnivorous bat species characterized by more leukocytes.^36^ Similarly, in Belize, neutrophil counts of a frugivore (*Sturnira parvidens*) decreased over time with land conversion, whereas those of hematophagous bats (*Desmodus rotundus*) increased and those of an insectivore bat (*Pteronotus mesoamericanus*) showed no response.^111^ To compare cellular immunity at a finer resolution, single‐cell RNA‐Seq has revealed different proportions of B cells in bone marrow and natural killer cells in the spleen between *Pteropus alecto* and *Eonycteris spelaea*.^112^, ^113^ Functional assays applied to bat sera samples have also found substantial interspecific differences in complement activity, with higher rates of lysis from *Eptesicus fuscus* than *Pteropus vampyrus*.^114^ Extensions of these baseline approaches have also revealed immune differences within genera; among sympatric horseshoe bat species in China, RNA‐Seq of organs found that *Rhinolophus siamensis* and *Rhinolophus episcopus* differ in the expression of immunoregulatory genes.^115^

Lastly, comparative genomics have emphasized the genetic basis of interspecific differences in bat immunity. Considering innate immunity, the composition of the type I IFN locus varies across bats, with initial work showing this locus is contracted in *Pteropus alecto* but expanded in *Pteropus vampyrus*, *Myotis lucifugus*, and *Rousettus aegyptiacus*.^31^, ^116^ Recent work has suggested IFN‐ω in bats may play an expanded antiviral role compared to other type I IFNs given that several bat species have lost all IFN‐α genes (i.e., *Pipistrellus kuhlii*, *Myotis myotis*, and *Pteronotus mesoamericanus*).^19^ Considering adaptive immunity, the immunoglobulin heavy chain (IGH) locus of bats is unusually variable between species. IGHV gene number varies substantially, with 132 genes in *Eptesicus fuscus*, 66 in *Rousettus aegyptiacus*, 41 in *Rhinolophus ferrumequinum*, 81 in *Phyllostomus discolor*, and 57 in *Pipistrellus pipistrellus*.^117^, ^118^, ^119^ In contrast, humans and mice possess 104 and 161 IGHV genes,^120^ respectively, and these species are over 60 million years further diverged than the most related bat species above (i.e., *Eptesicus fuscus* and *Pipistrellus pipistrellus*).^121^ Most strikingly, bats within the family Vespertilionidae possess two distinct and functional IGH loci,^117^ an organization that has not been previously described in mammals but bears similarity to a more limited duplication observed in teleost fish.^122^, ^123^

## EVOLUTIONARY HYPOTHESES IN BAT IMMUNOLOGY

As highlighted above, the pronounced diversity across bats is matched by substantial interspecific variation in immunity, as revealed by both experimental (e.g., Figure 2) and observational results. However, an outstanding need remains to identify the mechanisms underlying these species‐level differences. Here, we synthesize and propose hypotheses about the interspecific drivers of bat immunity: pathogen exposure, biogeography, host energetics, and environmental stability (Table 1). For each hypothesis, we present supporting research and outline potential directions for future studies. We note that while some trait drivers may lend themselves to testing a single hypothesis (e.g., pathogen richness to test hypotheses about pathogen exposure), others could shape immune diversity through multiple pathways (e.g., dietary diversity could test hypotheses about both pathogen exposure and host energetics).

**TABLE 1:** Proposed hypotheses that predict interspecific differences in bat immunology.

Mechanism	Driver	Prediction
Pathogen exposure	Coevolution	Host immune genes will show signatures of positive selection in response to pathogen pressure.
	Pathogen richness	Species with high pathogen diversity will invest more in adaptive immunity than those with few pathogens.
	Colony size	Species with large colonies will invest more in adaptive immunity if pathogens mainly follow density‐dependent transmission.
	Co‐roosting	Species that share roosts with more bat and nonbat species will invest more in adaptive immunity.
	Diet	Species that consume other animals should invest more in defense and have greater immunogenetic diversity.
	Habitat diversity	Greater habitat diversity (including large geographic range size and migratory distances) will promote immunogenetic diversity due to pathogen exposure.
	Longevity	Long‐lived species will invest more in adaptive immunity owing to accumulated pathogen exposure.
Biogeography	Speciation	Speciation events will correlate with diversification in immune strategies in both innate and adaptive arms.
	Genetic drift	Small and isolated populations will show reduced immune diversity due to drift and inbreeding.
Host energetics	Pace of life	Fast‐lived species will prioritize defenses with lower developmental costs (i.e., innate immunity).
	Diet	Species with low‐energy food will invest less in adaptive immunity than those with high‐energy food.
Environmental stability	Food seasonality	For species that do not hibernate or migrate, those with more seasonal food will invest more in innate defenses.
	Hibernation	Hibernating species will on average have lower baseline measures and weaker immune responses to conserve energy.
	Migration	Species with longer migrations between wintering and maternity grounds will show weaker immune responses.

*Note*: We qualify that such hypotheses are not mutually exclusive nor necessarily exhaustive.

### Pathogen exposure

One of the central hypotheses to explain immune variation among bat species focuses on the long coevolutionary history between chiropteran hosts and many of their pathogens. Across host taxa, pathogens impose strong selection pressures that can shape immunological diversity.^124^, ^125^ For example, pathogen richness is positively associated with major histocompatibility complex (MHC) variability across primate, ungulate, and a small number of bat species.^126^, ^127^ Bat–virus associations show strong signals of phylogeography that should also shape immune strategies. For example, henipaviruses are highly diverse in Africa, suggesting their likely origin in this region, and are primarily associated with pteropodid bats found only in Africa, Asia, and Oceania^128^, ^129^ (although serology has suggested henipa‐like viruses may circulate in select phyllostomids, restricted to the Americas^130^, ^131^). Likewise, bat‐associated filoviruses have only been found in Africa, Asia, and Europe,^132^ despite potential favorable host conditions in the Americas.^133^, ^134^ As one case study of immune adaptations structured by viral phylogeography, bats in the genus *Eidolon*, whose range includes the distribution of filoviruses, have a mutation in their host receptor that prevents EBOV entry.^94^ Similarly, influenza A viruses (IAVs) have been detected in diverse bats, including H17N10 and H18N11 from *Sturnira parvidens* and *Artibeus* species in the Neotropics as well as an H9N2‐like IAV from *Rousettus aegyptiacus* in Egypt.^135^, ^136^, ^137^ In the Afrotropical host, the H9N2‐like IAV preferentially binds to α2,3‐sialic acid receptors, while the Neotropical IAVs instead enter cells through the MHC class II DR protein;^138^ however, we note that such phylogeographic differences are complicated by the Neotropical IAVs originating from bat hosts, while the H9N2‐like IAV likely instead jumped from birds.^137^

Alongside expectations about coevolutionary histories shaping immunogenetics across bat species, pathogen diversity should also structure bat immune phenotypes. In other taxa such as birds, energetic investment into immune function is often elevated in areas of high pathogen richness (e.g., the tropics). For example, tropical bird species have more leukocytes in blood and larger spleen sizes than temperate bird species, with the latter indicating greater investment in adaptive immunity.^139^ Indeed, as antigen exposure drives the selection of specific cell populations and, in turn, the pool of B and T lymphocytes, greater exposure to pathogens should increase allocation to adaptive immunity.^140^ Explicit tests of how immunity is associated with pathogen richness across bats are needed to fully assess this hypothesis, which can be facilitated by standardized species‐level data on pathogen‐host status and diversity (e.g., VIRION; Figure 1).^75^

Multiple behavioral and life‐history traits of bat species could drive pathogen exposure, with subsequent effects on immune variation. For example, colony size varies several orders of magnitude across bats,^141^ with more colonial species possibly supporting pathogen transmission and thus investment into adaptive immunity. In birds, density‐dependent pathogen transmission in colonial species results in stronger B and T cell responses than in solitary species.^142^ However, support for density dependence in bat–pathogen systems is weak,^143^, ^144^ with exposure more likely a function of social and metapopulation structure or arthropod vectors.^145^, ^146^ Sociality may thus possibly have stronger effects on immunity via this exposure mechanism; in other mammals, more promiscuous species show greater investment in white blood cells, likely driven through increased exposure to sexually transmitted infections.^147^, ^148^ However, bat sociality is highly complex, with some species being characterized by seasonal maternity colonies^149^ or fission‐fusion societies.^150^ This complexity in social behavior will thus likely complicate efforts to understand how sociality drives species differences in immunity. Other interspecific differences in bat behavior, such as co‐roosting with other bat species, could also elevate pathogen exposure and have similar effects on interspecific variation in immunity.^151^, ^152^, ^153^, ^154^

**FIGURE 3:**
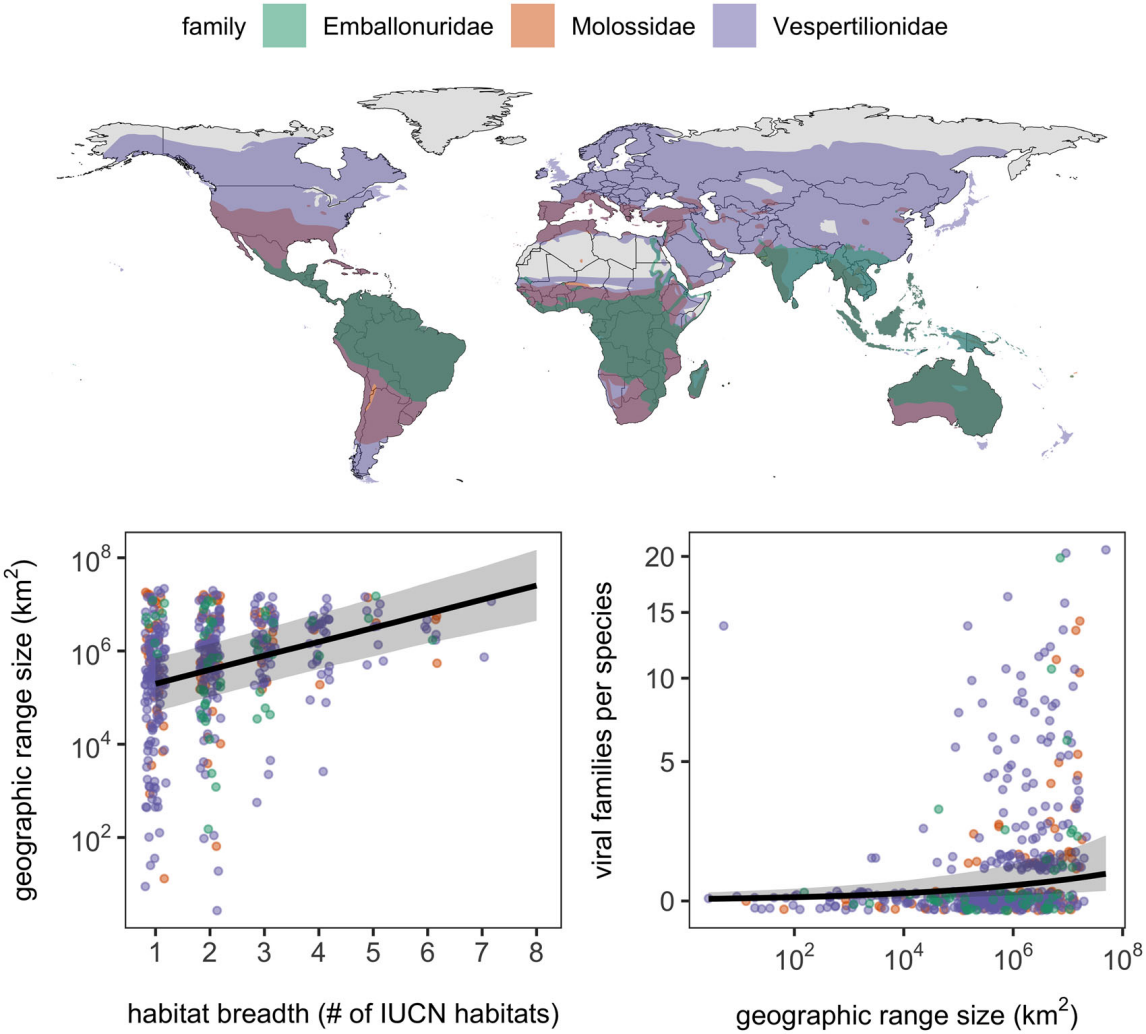
Geographic and behavioral distributions of global bat families: the Emballonuridae, Vespertilionidae, and Molossidae. Species distributions were drawn from the IUCN and merged per bat family using the *rgeos* R package. Inset plots show relationships between habitat breadth and geographic range size (left) as well as between geographic range size and viral family richness (right) across 516 species (not all species have matching trait data). Trait data are from COMBINE,^50^ PanTHERIA,^281^ and VIRION.^75^ Data are overlaid with the posterior mean slope and 95% credible interval (CI) band from each phylogenetic generalized linear model (PGLM) fit using the *brms* package,^282^ each using four chains including 2000 iterations and 50% burn‐in for a total 4000 samples (both models converged, given inspection of traces and $\hat{R}$ values). Within these three bat families, habitat breadth predicts log_10_‐geographic range size (β = 0.30, 95% CI: 0.21–0.39; PGLM with Gaussian response), and log_10_‐geographic range size predicts viral diversity (β = 0.37, 95% CI: 0.18–0.56) after adjusting for log total citation counts via the *easyPubMed* package (β = 0.93, 95% CI: 0.79–1.08; PGLM with negative binomial response). Viral family richness is displayed using a modulus transformation given the abundant zeroes.

The extreme dietary diversity observed across bats could also shape immune variation through pathogen exposure. Bat species that include more animals in their diets, particularly other vertebrates (e.g., phyllostomines including *Trachops cirrhosus*, *Chrotopterus auritus*, *Phyllostomus hastatus*, and *Vampyrum spectrum*; both *Noctilio* species; all three members of the Desmodontinae; *Myotis vivesi*; *Cardioderma cor*; *Megaderma lyra*; and *Macroderma gigas* ^155^), could be exposed to pathogens hosted by prey,^156^ selecting for greater investment in defense. Initial support for this hypothesis has been found within Neotropical bat communities using data on the cellular immune system.^36^ Other foraging‐related behaviors, such as large geographic ranges or high habitat breadth, as well as long‐distance migration, could also expose bats to a wider array of pathogens, as shown in birds^157^ and supported by select bat case studies (e.g., extreme MHC class I diversity in the geographically widespread *Carollia perspicillata* ^158^). In birds, migratory species invest more in immune organ size than resident species, supporting links between habitat diversity, pathogen exposure, and immunity;^159^ such comparisons have yet to be performed across bat species despite known variation in migratory strategies.^62^ Hypotheses about habitat breadth and geographic range more generally could be tested by comparing immunity among bat species in globally distributed taxa, such as the genus *Myotis* or several families (e.g., Figure 3). Lastly, longer‐lived species can accumulate pathogen exposure across their lifespan, as seen in birds, bats, and some terrestrial mammals,^160^, ^161^ which could also increase adaptive investment.

### Biogeography

Alongside coevolutionary history with pathogens, the distinct biogeography of many bats has likely contributed to their immunological diversity. Prior work on bat–CoV interactions has shown that regions with more evolutionarily distinct host communities harbor more divergent viral assemblages, which should likewise generate strong selective pressure for specialized immune adaptations.^78^ As one example, the historical biogeography of the Phyllostomidae and Pteropodidae resulted in their restriction to the Western and Eastern Hemispheres, respectively. Multiple gene families underwent expansion or contraction within the Pteropodidae, including those related to immunity, and this family has been characterized by the loss of the inflammasome *NLRP*1 gene and attenuated Toll‐like receptor 2 ability.^162^, ^163^ Similarly, genomic comparisons support the expansion of the *PRDM9* gene, which governs meiotic recombination and can be upregulated during viral infection in the Phyllostomidae compared to other bats.^19^ Further, the sister family Mormoopidae (also only in the Western Hemisphere) display major expansions of heat‐shock protein genes compared to other bats,^19^ indicating possibly unique adaptations involved in the stress response as well as in both innate and adaptive immunity.^164^

Recent work on the phylogenetic distribution of viral virulence also suggests biogeographic drivers in bat–pathogen interactions. Whereas previous work has found bats are more likely than other mammalian and avian orders to host viruses with high virulence in humans,^165^, ^166^ phylogenetic analyses agnostic to taxonomic order suggest that the Chiroptera do not emerge as a taxon more likely to harbor such viruses than other mammalian clades.^167^ Notably, a subclade of the Yangochiroptera consisting of the superfamilies Emballonuroidea and Vespertilionoidea was more likely to host high‐virulence viruses, with most included families being cosmopolitan (i.e., Emballonuridae, Vespertilionidae, and Molossidae; Figure 3). The shared ability to harbor otherwise virulent viruses in bat families that span both the Western and Eastern Hemisphere could suggest common immune adaptations that evolved with geographic divergence. For example, the Molossidae originated in the Paleocene, with Western (e.g., *Eumops*, *Molossus*) and Eastern Hemisphere (e.g., *Chaerephon*, *Mops*) clades diverging 29 million years ago.^168^ Future comparisons between species in the genus found globally (i.e., *Tadarida*) and between molossid genera unique to each hemisphere could indicate which immune features are basal to the family and which originated with the spread into the Americas.^168^

Biogeography could also shape bat immune diversity via differences in geographic range size. A smaller geographic range is one criterion used by the IUCN to delineate conservation risk since a lower effective population size can facilitate inbreeding depression and reduce genetic diversity.^169^ Species with smaller geographic ranges could, therefore, show less immunogenetic diversity (e.g., in MHC loci). Island occupancy could help test this hypothesis; over 25% of bat species are island endemic, and many have small population sizes and face critical extinction risks.^170^, ^171^ Immune comparisons of island endemic and nonendemic species in select bat genera (e.g., *Pteropus*, *Natalus*) or families (e.g., Pteropodidae) could thus be fruitful. From a similar perspective, subspecies that occur exclusively in islands could allow analogous comparisons among endemics and with mainland populations (e.g., within *Pteropus medius*, *P. medius medius* occurs in mainland India and Sri Lanka, while *P. medius ariel* occurs in the Maldives).^172^

### Host energetics

Different strategies in energy acquisition and allocation among bat species could affect immune investment, as developing and maintaining immune responses require substantial resources.^173^ Innate immunity generally incurs low developmental but high maintenance costs, while adaptive immunity can be more costly to develop but less expensive to maintain.^140^, ^174^ The pace‐of‐life hypothesis, therefore, posits that species with faster life histories, allocating more energy into reproduction at the expense of lifespan, will invest less into immunity and prioritize innate defenses.^175^, ^176^ In contrast, species with slower life histories and more likely to encounter similar pathogens multiple times over their lifespan invest more in adaptive immunity. While this hypothesis has been supported for some small mammals,^177^ it has yet to be evaluated for bats. Explicit tests of trade‐offs between innate and adaptive immunity among bat species that vary along the fast–slow axis are needed. Focusing such comparisons on females across species would be especially informative^178^ given the energetic costs of reproduction found in bats.^179^, ^180^

Similarly, diet can impose significant energetic constraints on bat species, influencing the trade‐offs observed between arms of the immune system.^181^, ^182^ Across phyllostomid bat species, nectarivores have a greater mass‐independent basal metabolic rate than other dietary guilds, although effects are sensitive when controlling for phylogeny.^183^ Similarly, strictly phytophagous species (e.g., in the Pteropodidae) have relatively less protein in their diet than other species, including frugivores or nectarivores with more flexible foraging strategies (e.g., *Glossophaga mutica* will actively hunt insect prey^184^) as well as strict insectivores or carnivores.^185^, ^186^ Links between high‐protein diets and investment in adaptive immunity are well‐established in model mammalian systems (i.e., humans and mice^182^) as well as in both domestic and wild birds,^181^, ^187^ although this has received little attention in bats.^188^, ^189^ Those bat species that rely on food with lower energetic content (e.g., obligate nectarivores and frugivores) are thus more likely to invest less in adaptive immunity when compared to species with energetically dense food (e.g., insectivores). Although this prediction mostly supports bat species at higher trophic levels investing more in adaptive defense, blood‐feeding species (i.e., Desmodontinae) could serve as an exception owing to their unique diet of blood, which is high in protein but lacking in other macronutrients.^190^ The low‐fat content of blood likely led to the loss of genes governing fat storage in vampire bats,^191^ such that these species lethally starve within 72 h of feeding.^192^, ^193^ The ability to invest in adaptive defenses may thus be diminished in blood‐feeding bats. Given the importance of lipids in immune defense more generally,^181^, ^182^, ^187^ interspecific differences in fat reserves could serve as another useful source of dietary variation to test energetic hypotheses.^194^

### Environmental stability

Lastly, bat species inhabiting environments with more extreme seasonality in resources or climate, such as temperate zones or high elevations, could similarly differ in their ability to invest in immune defense. Periods of limited food availability could weaken the acute phase response^195^ as well as immune factors that control pathogen shedding^189^, and thus manifesting in differences at the species level among bats that have seasonally varying versus stable resources. As one example relevant to immunity, seasonal patterns of cortisol concentrations differed between frugivorous *Carollia perspicillata* and blood‐feeding *Desmodus rotundus*, likely driven by differences in resource stability.^196^ Yet, while seasonality in resources is particularly evident in phytophagous and insectivorous bat species,^197^, ^198^, ^199^ food availability can vary temporally across dietary guilds,^200^ such that these effects could be tested independently from foraging ecology. Given the relative costs of the two primary immunological arms noted above, bat species with more seasonal resources could also be expected to thereby invest more in innate defenses.^140^, ^201^

Prolonged torpor or hibernation function as other strategies that bat species use to cope with environmental instability,^202^ which could also generate interspecific variation in immune strategies. These pronounced reductions in metabolic activity and body temperature allow such species to conserve energy but at the cost of a dampened innate and adaptive immune response.^203^, ^204^ Impaired immunity during hibernation can have important implications for susceptibility and persistence of infection. For example, lowered body temperature during hibernation and downregulation of immune response can extend the incubation period of RABV in North American bats^205^, ^206^ and likely allows the virus to overwinter and persist in the spring when bats emerge from hibernation.^207^ Similarly, *Myotis myotis* cell lines challenged with the RABV‐related European bat lyssavirus 1 showed an immune response under control conditions but no substantial immune gene expression under conditions simulating torpor.^208^ Interspecific differences in torpor could thus serve as an important axis for partitioning immune variation,^57^ with particular relevance for susceptibility to and progression of WNS. Arousal from torpor contributes to the depletion of fat stores and in turn the severity of infection, although inflammatory responses during arousal also play a role in pathology.^209^, ^210^ Importantly, because immune responses to fungal infection display variation among bat species,^211^, ^212^ future work evaluating how interspecific differences in torpor duration and body temperature affect immune responses could be highly relevant for both hypothesis testing and conservation management.

Seasonal migrations offer select bat species another approach to deal with seasonally varying temperatures or resources. Short‐ and long‐distance migrations occur across the bat phylogeny but are especially concentrated within Vespertilionidae and Molossidae.^61^, ^62^ Across taxa, migratory species often redistribute resources from their immune systems to increase body fat and enhance metabolism prior to these long‐distance movements as these physiological changes sustain endurance.^213^ Work in avian systems supports the suppression of immune function prior to, during, and/or following migration,^214^, ^215^ with consequences for enhancing susceptibility to or reactivation of infections.^216^, ^217^ By contrast, research on the immunology of migratory bat species is still in its infancy.^218^, ^219^, ^220^ Future work comparing immune phenotypes of migratory and nonmigratory species, as well as species varying in their migratory strategies (e.g., average distance traveled), would test whether similar patterns of immunosuppression are observed within bats. Similarly, comparisons among subspecies that vary in their propensity to migrate (e.g., partially or fully migratory *Tadarida brasiliensis mexicana* vs. resident *T. brasiliensis cynocephala* ^221^) would also be informative. Variation in mean migratory distance and dispersion among bat species, measures commonly used in comparative avian studies,^222^, ^223^ could especially allow testing hypotheses of energy allocation given that species with longer migrations should display weaker immune responses.

## FUTURE DIRECTIONS FOR ILLUMINATING SPECIES‐LEVEL DIFFERENCES IN BAT IMMUNITY

Current hypotheses on the drivers of interspecific variation in bat immunity (Table 1) are supported by select case studies as well as first principles in host–pathogen coevolution and ecological immunology. To robustly test and differentiate these competing hypotheses, the field of bat immunology must address outstanding data needs, methodological advancements, expansion of experimental studies, and phylogenetically informed statistical analyses.

First, broad sampling across bat species is essential to better characterize the diversity of immune components, function, and response to infection. To date, comparative tests of bat immunity have largely been limited to genomic comparisons or to analyses of phenotypes within single bat communities,^25^, ^36^ with some exceptions.^224^ At the genomic level, ongoing efforts are working to generate genome assemblies across bat species (e.g., the Bat1K Project),^225^ and resulting comparative analyses have provided important insights into bat evolution (including the immune system).^15^, ^25^, ^226^, ^227^, ^228^, ^229^ However, of the currently recognized 1487 bat species, genome assemblies are currently publicly available at the National Center for Biotechnology Information (NCBI) for only 92 species (Table S1). Further, only 47 of these species have chromosome‐level assemblies, which are often required to properly characterize complex immune gene loci.^117^, ^231^ Additionally, while these genomes are invaluable resources, characterizing the diversity of bat immune systems requires a more systematic evaluation of downstream phenotypes. For example, while genomic data indicate *Pteropus alecto* has a small type I IFN locus, qRT‐PCR data show IFN‐α is instead constitutively expressed.^31^ Similar tests are needed across more bat species.

**FIGURE 4:**
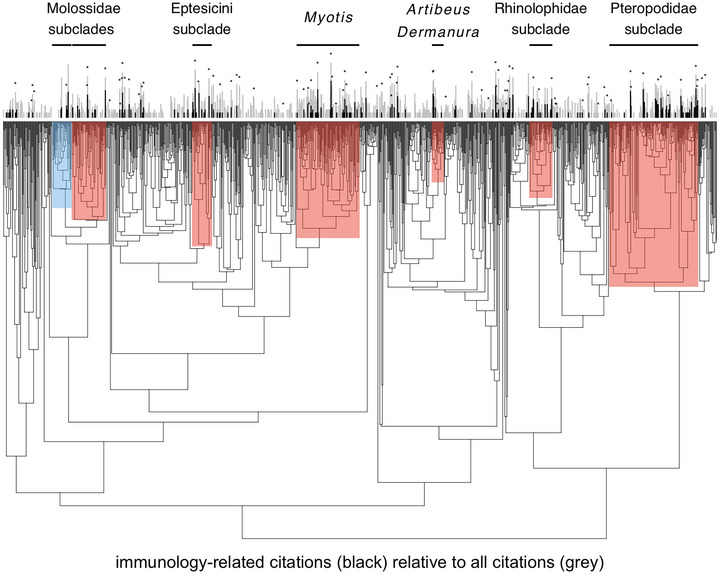
Taxonomic patterns in the relative number of immunology‐related citations and public availability of genome assemblies per bat species (Table S1), as two measures of data coverage. The phylogeny (*n* = 1287 species)^231^ is presented with seven clades identified from phylogenetic factorization of immunology citations, modeled with the *phylofactor* package in R as a binomial response to account for the total citation count per species.^233^ Clades with proportionally greater or fewer immunology citations compared to the rest of the phylogeny are shown in red and blue, respectively, with segments showing the raw counts of total citations (gray) and immunity citations (black). Asterisks are provided for those species with an NCBI genome assembly.

On a more general level, data syntheses of bat immunology as a field are lacking, resulting in a limited understanding of how research is distributed across the bat phylogeny. To provide an initial characterization of immunological studies conducted across bats, we used the *easyPubMed* package in R to obtain total and immunology‐related citation counts for the 1287 bat species in the recent mammalian phylogeny;^231^ citation counts are a common approximation of research effort in comparative analyses.^232^ Search strings contained either bat genus and species (e.g., *Desmodus* AND *rotundus*) or bat genus, species, and two stems to capture the immune system (i.e., immuni* OR immunolog*); strings used Latin binomials from the phylogeny.^231^ Despite the fact that most bats have been studied to some degree (i.e., 55% of species have greater than zero total citations), only 14% of bats have immunology‐related citations (Figure 4). To understand the taxonomic distribution of research effort, we next applied phylogenetic factorization, a flexible graph‐partitioning algorithm, to identify bat clades with distinct citation counts at varying taxonomic depths.^233^ We used the *phylofactor* package to partition immunology‐related citations relative to total citations as a binomial response in a series of generalized linear models for each edge in the bat phylogeny, determining the number of significant clades using Holm's sequentially rejective 5% cutoff for the family‐wise error rate.^233^, ^234^ We identified seven clades with significantly different numbers of immunology‐related citations, of which six had more immunology citations compared to the remainder of the bat phylogeny (Figure 4). These clades included most of the Pteropodidae; a subclade of the Rhinolophidae; most members of the genus *Tadarida* and the Western Hemisphere molossids; a subclade of the tribe Eptesicini; the whole genus *Myotis*; and the clade containing the genera *Artibeus* and *Dermanura*. In contrast, Eastern Hemisphere molossids (e.g., the genera *Mops* and *Chaerephon*) had relatively fewer immunology citations. This assessment highlights the substantial gaps in immunological characterization across bats as a whole, noting clades that could be up‐ or down‐prioritized for future immune profiling (e.g., Afrotropical molossids and most pteropodids, respectively). In contrast, the application of this algorithm to the presence of NCBI genome assemblies showed no phylogenetic clustering (Figure 4), suggesting that genomic characterization efforts to date have been evenly distributed across bat species.

**FIGURE 5:**
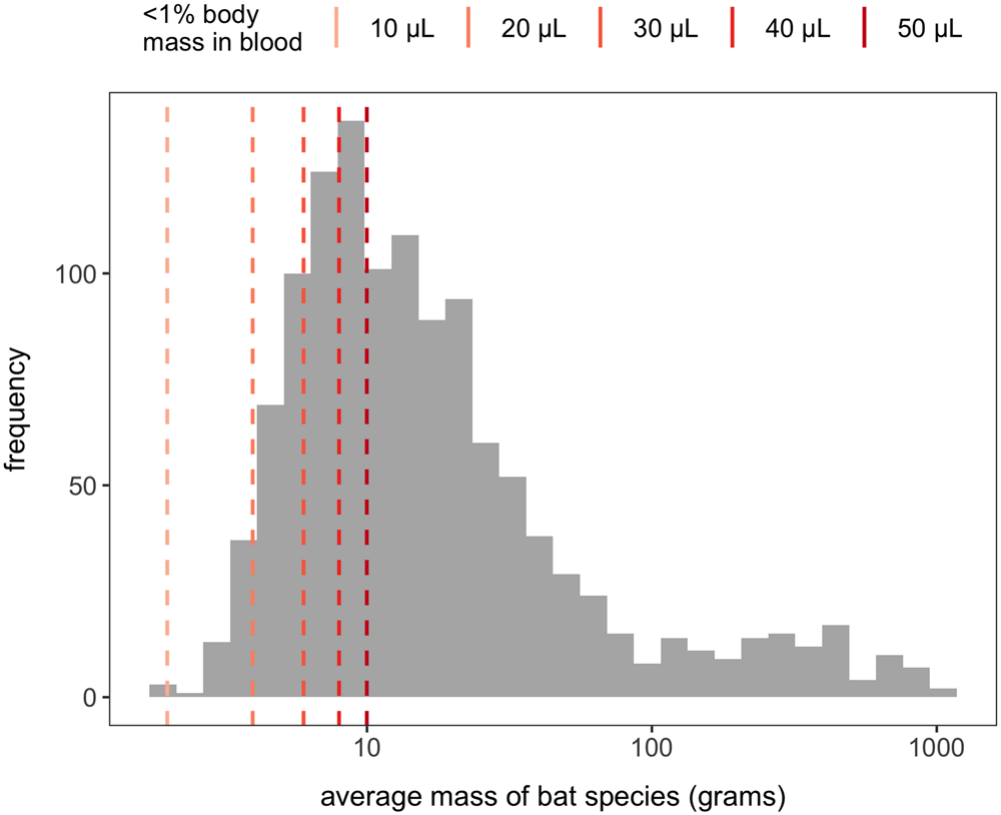
Distribution of average body mass across bat species (*n* = 1217).^50^, ^232^ Overlaid are the minimum body masses for which varying small blood volumes can be safely and nonlethally obtained (representing approximately 0.5% body mass as a highly conservative limit).^283^, ^284^

Second, methodological expansion is necessary to better characterize immunological variation across bat species and fill these global data gaps. In wild bats, relatively simple assays such as total and differential white blood cell counts, bacterial killing ability of plasma, and antibody titers have provided key starting points to profile bat immunity.^36^, ^219^, ^235^, ^236^ However, these assays require most of the small blood volumes that can be safely obtained from the majority of bat species (Figure 5), limiting the number of assays that can be performed while yielding information on single components of the immune system. Further, the coarse nature of these measurements and the lack of knowledge about prior or existing immune challenges in wild bats also restrict mechanistic insights into immunity. Flow cytometry holds promise for quantifying many immune cell subsets beyond that allowed by typical hematology, but analyses remain restricted by the larger blood volumes required, the need to process samples relatively soon after collection, and the limited availability of cross‐reactive antibodies for bats.^112^, ^237^, ^238^, ^239^ Alternatively, the increasing adoption of ‐omics approaches can investigate hundreds or even thousands of immune components at once (e.g., transcripts, proteins) without species‐specific or cross‐reactive reagents. In particular, proteomics can provide data on hundreds of proteins from very small volumes of plasma or sera, making the most of the limited samples nonlethally obtained from wild bats.^240^, ^243^ Single‐cell RNA‐Seq can facilitate flow cytometry analyses through antibody‐independent identification of cell types and further facilitate the study of biological processes in heterogeneous cell populations. This method has been applied to several bat species.^76^, ^113^, ^239^, ^243^ However, costs can still be prohibitive depending on the scale of the experiment.^244^ Between more historic and newly applied methodologies for characterizing the immunity of wild bats especially, an outstanding need is the development of comparable and accessible protocols for collecting and storing biological samples, conducting assays, and analyzing raw data to standardize approaches and enable comparisons among studies.

Third, the expansion of experimental studies will be central to advance the tools used in bat immunology and to mechanistically test evolutionary hypotheses. Increased representation of major bat families in captive systems is needed to develop bat‐specific immunological tools,^30^ including but not limited to monoclonal antibodies.^238^ Such captive systems will be especially important for better characterizing and comparing parts of bat immunity that remain elusive, such as adaptive defense.^10^, ^117^, ^245^ Several studies have shown variation in the B cell and antibody response among bat species,^99^, ^100^ although the drivers behind these differences remain poorly understood. Studies have also focused on neutralizing antibodies, such that our understanding of other aspects of the humoral immune response, including the role of non‐neutralizing antibodies and Fc receptor functions, likewise remains limited. However, given the challenges associated with maintaining captive bat colonies,^246^, ^247^ greater adoption of in vitro models should especially enhance mechanistic insights into the patterns of immunity and infection observed in the wild. For example, the persistence of a novel α‐CoV was observed in *Myotis lucifugus* for at least 4 months during hibernation without detectable pathology.^248^ Infection of cell lines derived from another vesper bat, *Eptesicus fuscus*, with MERS‐CoV recapitulated this duration of viral persistence but further demonstrated that this phenomenon was associated with an IFN regulatory factor 3–dependent antiviral response.^249^ Organoid models in particular could be especially informative given their ability to model whole immunological tissue.^87^, ^90^, ^92^, ^250^ Immunological differences in wild bat species could then be interrogated with more focused, controlled tests in these in vivo and in vitro models (e.g., via mock or actual infection between bat species).^251^

Finally, the application of phylogenetic comparative methods and other statistical tools are central to test support for the correlated evolution of bat species traits and immunological outcomes. Phylogenetic generalized linear models (PGLMs) or phylogenetic generalized linear mixed models (PGLMMs) should be a primary approach to control for evolutionary history, depending on the use of species‐level (i.e., mean or binary immune outcomes) or individual‐level data, respectively. For PGLMs, weighting strategies can account for variation in sample size or levels of precision in species means and provide more robust estimates of model coefficients and the ability to test hypotheses.^252^, ^253^ For PGLMMs, including both phylogenetic and nonphylogenetic species, random effects can reduce bias and improve inference.^254^, ^255^ Other statistical methods, including but not limited to ancestral state reconstruction, state‐dependent diversification, and phylogenetic factorization, would facilitate improved understanding of the evolution of bat immune systems, their relation to speciation and extinction, and identify distinct lineages of immune strategies.^167^, ^222^, ^256^ Collectively, this suite of analyses has been applied to comparative immunology studies of other vertebrate taxa,^222^, ^257^, ^258^, ^259^, ^260^, ^261^, ^262^, ^263^, ^264^ and addressing immunological data gaps across bat species (Figure 4) will enable greater adoption of these methods to the Chiroptera. Study of understudied bat species will also confront sparsity in and robustness of trait data,^265^, ^266^, ^267^ including pathogen diversity and coevolutionary histories (e.g., via phylogenetic dating).^268^, ^269^

**FIGURE 6:**
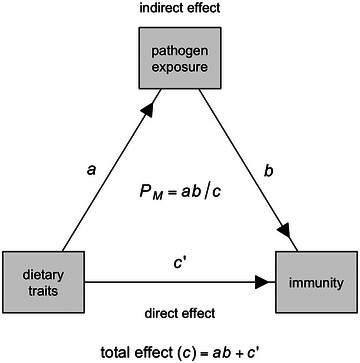
Example of how causal mediation analysis (CMA) can differentiate hypotheses about the drivers of interspecific variation in bat immunology by considering alternative mechanisms of pathogen exposure and host energetics. Here, CMA estimates the total indirect relationship between a dietary trait and pathogen exposure (*a*) and pathogen exposure and immunity (*b*) as well as the direct relationship between a dietary trait and immunity (*c’*). The total effect (*c*) is then the sum of the indirect effect (*ab*) and the direct effect (*c’*). The proportion mediated by pathogen exposure (*P_M_*) is derived as the indirect effect (*ab*) divided by the estimated total effect (*c*): *ab*/*c*. High estimates of *P_M_* support the indirect relationship (i.e., pathogen‐mediated selection), whereas negligible *P_M_* estimates better support the direct relationship between diet and immunity (i.e., host energetics).

To statistically differentiate multiple, competing evolutionary hypotheses about the drivers of interspecific variation in bat immunology, we suggest greater adoption of frameworks for causal inference,^270^, ^271^ such as causal mediation analysis (CMA).^272^ Similar to structural equation modeling, CMA decomposes a hypothesized causal relationship between a predictor and a response into the direct effect and the indirect effect mediated through a third variable. This approach could be especially useful in cases where a given trait driver is hypothesized to affect immunity through multiple mechanisms, such as for diet (Table 1). Here, CMA would estimate the direct effect of diet on immunity (representing energetic hypotheses) as well as the indirect effect of diet on pathogen exposure (Figure 6). Importantly, PGLMs or PGLMMs can be used in these analyses, and both the mediator and outcome models can adjust for relevant precision covariates, such as citation counts (i.e., for species‐level analyses) or time between capture and blood collection (i.e., for individual‐level analyses). Controlling for such variables, especially those well‐known to introduce artifacts into immunology data,^273^, ^274^ will more generally be important for accurate estimation of effects when testing evolutionary hypotheses.

## CONCLUSION

A robust body of work has identified distinct mechanisms by which the immune systems of bats differ from other mammals, with downstream consequences for how chiropteran hosts resist or tolerate virulent infections. Yet, as we have highlighted in this review, the order Chiroptera is not a monolith. The pronounced ecological and evolutionary diversity observed across bat species also corresponds to notable heterogeneity in immune strategies. We have here proposed multiple, nonmutually exclusive hypotheses to explain this interspecific variation in bat immunity seen observed to date; testing and differentiating these will require confronting key sampling gaps, capitalizing on methodological advancements, integrating in vitro and in vivo studies, and adopting phylogenetically informed statistical analyses. Ultimately, such work will advance our understanding of the drivers and consequences of immunological diversity among bats. At the same time, given the upward momentum in research on bat immune systems,^10^, ^30^, ^275^, ^276^ the efforts we have proposed here could have profound follow‐on effects for studying and understanding the diversity and evolution of immune systems across vertebrate hosts more generally.^125^, ^277^, ^278^

## AUTHOR CONTRIBUTIONS

D.J.B. and H.K.F. conceptualized the paper; D.J.B., A.V.S., A.B.R., B.R.A., M.O., C.A.C., A.J.R., R.M.Q.‐T., M.M.T.P., J.R., A.B., and H.K.F. outlined and wrote the paper; and D.J.B. analyzed data, generated figures, and edited and revised the manuscript. All authors approved the final version.

## COMPETING INTERESTS

The authors declare no conflicts of interest.

## PEER REVIEW

The peer review history for this article is available at https://publons.com/publon/10.1111/nyas.15395.

## Supporting information



Supporting Information

## Data Availability

Species‐level data used in Figures 1, 3, 4, and 5 are provided in the Dryad Digital Repository (https://doi.org/10.5061/dryad.ksn02v7gj). While COMBINE, PanTHERIA, and the mammal phylogeny are static datasets, we caution that host–virus associations and citation data from VIRION and PubMed, respectively, are dynamic and prone to change.^268^ We thus provide an R script to aggregate virus data from VIRION and citation count data via the *easyPubMed* package.
